# Changes in the distribution of endogenous hormones in *Phyllostachys edulis* ‘Pachyloen’ during bamboo shooting

**DOI:** 10.1371/journal.pone.0241806

**Published:** 2020-12-11

**Authors:** Zhan Shen, Yan-hua Zhang, Lei Zhang, Yuan Li, Ya-dong Sun, Zu-yao Li

**Affiliations:** 1 College of Forestry, Jiangxi Agricultural University, Nanchang, Jiangxi, China; 2 Jiangxi Provincial Key Laboratory for Bamboo Germplasm Resources and Utilization, Nanchang, Jiangxi, China; 3 College of Forestry, Nanjing Forestry University, Nanjing, Jiangsu, China; Michigan State University, UNITED STATES

## Abstract

In this study, we investigated the changes in the distribution and regulation of endogenous hormones in *Phyllostachys edulis* ‘Pachyloen’ during bamboo shooting. Enzyme-linked immunosorbent assay was used to measure the mass fractions of indole-3-acetic acid (IAA), gibberellic acid (GA), zeatin riboside (ZR), and abscisic acid (ABA) in rhizomes, shoots, and maternal bamboo organs during shoot sprouting, shoot growth, and new-bamboo formation. Measurements were compared among bamboo parts and developmental periods. The overall mass fractions of IAA and ABA were significantly higher than those of ZR and GA, driven by differences among bamboo parts and developmental periods. The abundance of each endogenous hormone varied among bamboo parts and developmental periods. During bamboo shooting, ABA had the highest mass fraction in all bamboo parts sampled, followed by IAA, GA, and ZR. Among bamboo parts, rhizomes had more IAA, ZR, and GA than the other parts, but significantly less ABA. Winter shoots had higher ZR: IAA and GA: IAA ratios than rhizomes and maternal bamboo organs. During shoot growth, ABA was the most abundant hormone in rhizomes and maternal bamboo organs, followed by IAA, ZR, and GA. In contrast, IAA was the most abundant hormone in spring shoots, followed by ABA, ZR, and GA. Maternal bamboo organs had a significantly higher ZR: GA ratio, and significantly lower IAA: ABA, ZR: ABA, and GA: ABA ratios than rhizomes. Spring shoots had significantly higher IAA: ABA, ZR: ABA, and GA: ABA ratios than rhizomes and maternal bamboo organs; significantly higher ZR mass fractions, and ZR: GA and ZR: IAA ratios and significantly lower ABA mass fractions than rhizomes; and significantly higher GA: IAA ratio than maternal bamboo organs. During new-bamboo formation, ABA was the most abundant hormone in rhizomes, winter shoots, and maternal bamboo organs, followed by IAA, ZR, and GA. Maternal bamboo organs had significantly lower IAA mass fractions and significantly higher ABA mass fractions than rhizomes and new bamboo tissue. IAA and ABA abundances exhibited an inverse relationship in rhizomes and maternal bamboo organs. GA: ABA and GA: IAA ratios decreased gradually and other hormone ratios exhibited parabolic trends over the bamboo-shooting period, with the highest ratios observed in new bamboo tissues. Overall, the coordination or antagonism among endogenous hormones plays a key regulatory role in bamboo shoot growth. The formation of thick walls in *P*. *edulis* ‘Pachyloen’, one of its major traits, may be partially attributed to the relatively high IAA and ZR and low GA mass fractions.

## 1. Introduction

*Phyllostachys edulis* ‘Pachyloen’ is a unique bamboo variety found in Jiangxi Province, China. It was the first bamboo variety in China to obtain protection under legislation for new plant varieties in 2008 [1], and was certified as a “National Fine Variety” in 2017. The stalk wall of *P*. *edulis* ‘Pachyloen’ is 1.8 to 2.0 times thicker than that of moso bamboo with similar stalk diameters. This wall thickness is considered a stable trait [2]. Generally, the biological and ecological characteristics of *P*. *edulis* ‘Pachyloen’ are similar to those of moso bamboo. However, the shoots of *P*. *edulis* ‘Pachyloen’ have a higher yield and nutrient content in terms of proteins, amino acids, and crude fiber [3]. Furthermore, *P*. *edulis* ‘Pachyloen’ is colder tolerant [4], and has higher nitrogen metabolic and use rates [5] and higher rates of carbon dioxide and light energy uptake [6, 7]. Because *P*. *edulis* ‘Pachyloen’ is used for both shoot consumption and timber, it has a high economic value, and there is significant research interest in its germplasm and carbon storage and sequestration capacity [8, 9].

The levels of endogenous hormones and their interactions help to regulate plant growth and development [10, 11]. Studies have investigated endogenous hormones in Lei bamboo [12, 13] and in germinating bamboo seeds using cobalt-60 irradiation [14]. The distribution and characteristics of endogenous hormones were also studied in maternal bamboo plants and bamboo shoots during the shooting period [15, 16], in bamboo stalks during the leaf-expansion period in *P*. *edulis* ‘Pachyloen’ [17], and in response to bamboo reproduction in *P*. *edulis* ‘Pachyloen’ [18]. Furthermore, Wu et al. [19] studied the effects of chloramphenicol on pigment mass fractions and chlorophyll fluorescence in moso bamboo seedlings. The results from these studies show that endogenous hormones greatly influence the growth and development of bamboo shoots. In addition, the mass fractions of endogenous hormones vary among bamboo organs and parts, and are affected by human interventions.

Studies on endogenous hormones in bamboo have typically focused on a specific developmental period or organ, and on the mass fraction and distribution of a single hormone. However, additional information on the changes in distribution of endogenous hormones in the entire bamboo forest during bamboo shooting is needed. Specifically, studies are needed to clarify how endogenous hormones in bamboo rhizomes, shoots, and maternal organs respond to changes in growth activity between the aboveground and belowground bamboo systems, and how endogenous hormones interact in synergistic and antagonistic ways to regulate shoot growth.

Therefore, in this study, we measured the mass fractions of endogenous hormones in bamboo rhizomes, shoots, and maternal organs at different developmental periods—from rhizome sprouting to bamboo growth—to analyze the changes in distributions of endogenous hormones during the shooting process in bamboo forests, elucidate the regulation of endogenous hormones, and investigate the effects of synergistic or antagonistic interactions among hormones on bamboo shooting. We aim to clarify how endogenous hormones regulate the growth and development of bamboo plants such as *P*. *edulis* ‘Pachyloen.’ Our results provide a theoretical basis for using exogenous hormones to promote bamboo shooting in *P*. *edulis* ‘Pachyloen’ and direct the cultivation of bamboo forests.

## 2. Materials and methods

### 2.1 Study site

The study site is located at the Bamboo Plant Germplasm Park of Jiangxi Agricultural University, China (28°45′24″N, 115°49′50″E). The landscape consists of low hills with a mean elevation of 49.5 m. The climate is mild and humid with sufficient sunshine for bamboo growth. The annual mean temperature is 17.0–17.9°C, and the annual maximum and minimum temperatures are 40.9 and −15.2°C, respectively. Mean annual rainfall is 1600–1700 mm, which is typical of a subtropical monsoon climate. The study site has red soil that is viscous and acidic.

### 2.2. Sample collection

Samples were obtained from a pure stand of *P*. *edulis* ‘Pachyloen,’ which was grown from maternal bamboo transferred in 1995 from Yantian village, Gaocun township, Wanzha county, Jiangxi province, China (28°06′33.38″N, 114°26′25″E), the origin of this bamboo variety. The area of the stand was approximately 0.5 ha. The diameters of the bamboo stalks in the stand were 4.0–7.0 cm, and bamboo heights were 6.0–10.0 m. The bamboo plants were closely spaced and exhibited vigorous growth.

Samples were first collected on January 20, 2015, when bamboo shoots were sprouting. We sampled 3-year-old maternal bamboo, 4–5-year-old rhizomes, latent buds on rhizomes (unterminated rhizome buds), new shoots, and winter shoots. Samples were collected again on March 31, 2015, when bamboo shoots were growing. This time, we sampled 3-year-old maternal bamboo, 4–5-year-old rhizomes, latent buds on rhizomes, and spring shoots. Finally, on June 10, 2015, during the period of new-bamboo formation, we sampled 3-year-old maternal bamboo, 4–5-year-old rhizomes, latent buds on rhizomes, and new bamboo plants.

**Fig 1:**
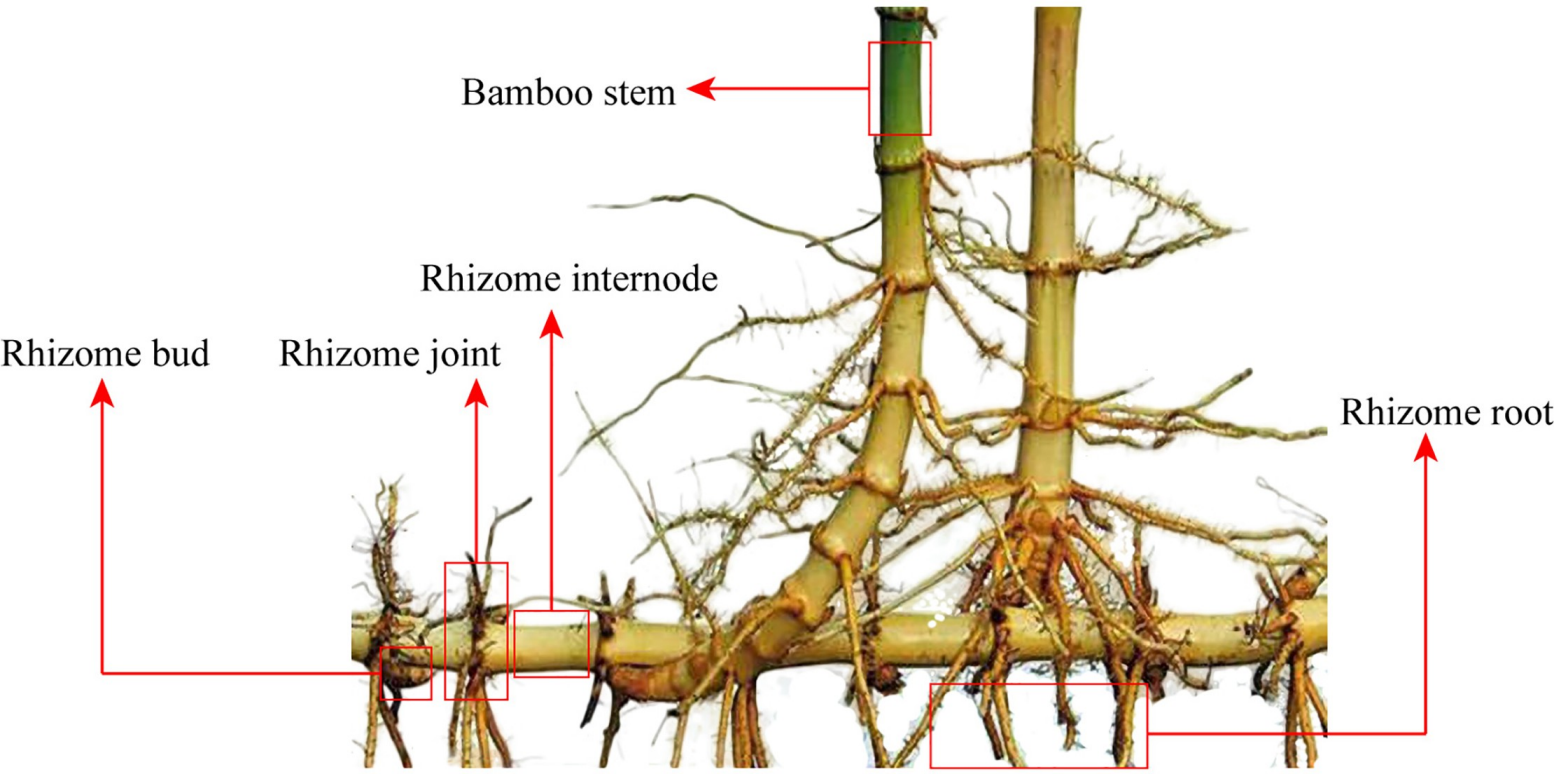
The organs of bamboo rhizomes.

In total, we collected 22 bamboo rhizomes: 14 from the shooting period, 3 from the shoot-growing period, and 5 from the period of new-bamboo formation. Each rhizome was divided into the internode, joint, root, and latent bud. The organs of bamboo rhizome were shown in Fig 1. We also sampled the stem, branches, leaves, and roots of nine maternal bamboo plants (three in each sampling period). Whole latent buds were collected three times in each sampling period. During bamboo shooting, three 3-cm shoot buds and three 10-cm winter shoots were collected. Spring shoot buds were divided into buds and sheaths, and winter shoots were divided into shoot tips, centers, bases, and sheaths. During the shoot growing period, four 30-cm shoots were collected and divided into shoot tips, centers, bases, winter sheaths, and roots. During the period of new-bamboo formation, three new bamboo plants were collected and divided into stems, branches, leaves, and roots. Each sample weighed approximately 1 g.

### 2.3. Sample processing

After samples were collected in the field, they were quickly wrapped in tin-platinum paper, placed in an ice box, and transported to the laboratory for cryopreservation at −85°C in an ultra-low-temperature freezer. Weighed 0.2–0.5g sample, added 4mL 80% methanol solution (containing di-tert-butyl p-cresol 1 mol/L), extracted at 4°C for 8h, and then centrifuged at 4000r/min for 10min. Took the supernatant for precipitation, then repeated with 80% methanol extract for 2 times. Combined the supernatants, and purifyed by Sep-Pack C18 column. Dried with nitrogen and dissolved PBSTG to a constant volume of 2mL.

### 2.4. Determination of endogenous hormones

The mass fractions of indole-3-acetic acid (IAA), gibberellic acid (GA), zeatin riboside (ZR), and abscisic acid (ABA) were determined using enzyme-linked immunosorbent assay. Each sample was measured in parallel three times. The specific method were as follows:

1) Marked the position corresponding to the sample to be tested on the plate well. 2) Manually cleaned the plate: drained the liquid in the well, filled each well with the cleaning liquid, let it stand for 1 minute, and then drained the liquid in the well. Patted dry on absorbent paper, then washed the plate for 3 times. 3) Set standard wells and sample wells on the wells of the plate, and added 50μL of standard solutions of different concentrations to each well. 4) Added 10μL of the sample to be tested to the sample well, then added 40μL of sample diluent. 5) In addition to the blank holes, added 100μL of horseradish peroxidase (HRP)-labeled detection antibody to each of the standard wells and sample wells, sealed the reaction wells with a sealing film, and incubated at 37°C for 60 minutes. 6) Discarded the liquid, patted dry on absorbent paper, filled each well with washing liquid, and let stand for 1 minute. Shook the washing liquid, put it on absorbent paper, patted dry, and repeated 5 times. 7) Within 15 minutes, added 50μL of stop solution to each well, and measured the absorbance of each well using a microplate reader with a wavelength of 490 nm. 8) Result calculation: The abscissa of the standard curve was represented by the natural logarithm of each concentration of the hormone standard sample, and the ordinate was represented by the logit value of the color value of each concentration. The logit value was calculated as follows:

$$Logit\;(\frac{B}{B_{0}})=ln\frac{B}{B_{0}-B}$$

Where B_0_ was the color value of 0ng/ml well, and B is the color value of other concentrations.

The natural logarithm of the hormone concentration contained in the test sample could be found from the standard curve on the graph according to the logit value of its color rendering value, and the hormone concentration could be obtained through the antilog. After obtaining the concentration of the hormone, calculated the content of the hormone in the sample.

### 2.5. Data processing

Data were pre-processed using Microsoft Excel 2016 (Microsoft Corporation, Redmond, WA, USA) and analyzed using SPSS software (v. 24.0; IBM Corp., Armonk, NY, USA). One-way analysis of variance and Duncan’s new multiple range test were used to compare the mass fractions and ratios of IAA, GA, ZR, and ABA among developmental periods and bamboo parts.

## 3. Results

### 3.1. Distribution of endogenous hormones during the shooting period in *P*. *edulis* ‘Pachyloen’

#### 3.1.1 Shoot-sprouting period

The mass fractions of endogenous hormones in maternal organs, rhizomes, and shoots during the shoot-sprouting period are displayed in Table 1.

**Table 1:** Mass fractions of endogenous hormones in different bamboo parts during the shoot-sprouting period.

Bamboo part	Organ	IAA (ng·g^−1^)	GA (ng·g^−1^)	ZR (ng·g^−1^)	ABA (ng·g^−1^)
Maternal organs	Leaf	58.51±24.82 Aab	11.17±3.35 Aa	5.42±0.94 Aa	162.66±113.26 Ab
	Branch	73.82±37.17 Aab	10.56±3.61 Aa	5.18±1.76 Aa	145.35±108.29 Ab
	Stem	67.16±41.79 Aab	10.63±0.34 Aa	5.61±1.25 Aa	139.76±90.61 Ab
	Root	62.39±14.78 Ab	13.96±3.8 Aa	5.84±0.38 Aa	69.2±43.06 Ab
Rhizomes	Rhizome internode	71.16±26.76 Ab	15.05±9.14 Aa	7.05±2.89 Aa	80.12±42.75 Ab
	Rhizome joint	70.39±24.87 Ab	15.95±10.87 Aa	7.14±3.13 Aa	84.64±41.52 Ab
	Rhizome root	79.63±27.04 Ab	19.15±9.25 Aa	7.70±3.36 Aa	72.27±47.21 Ab
	Latent bud	78.73±29.60 Ab	17.54±10.31 Aa	6.90±2.62 Aa	79.32±34.54 Ab
Shoots	Shoot bud	64.84±9.25 Ab	6.6±2.66 Aa	11.35±4.22 BCa	84.23±14.07 Ac
	Spring shoot	52.09±5.24 Ab	5.01±1.26 Aa	11.95±3.22 Ca	103.01±32.97 Ac
	Sheath of winter shoot	41.43±24.9 Aab	10.64±0.78 Aa	4.84±0.68 Aa	86.84±49.84 Ab
	Winter shoot	53.32±23.99 Aab	15.81±6.91 Aa	7.18±3.18 ABa	116.44±73.39 Ab

Note: Different uppercase letters represent significant differences (p < 0.05) among different organs of the same bamboo part. Different lowercase letters represent significantly different levels (p < 0.05) among hormone types in the same organ. IAA: indole-3-acetic acid, GA: gibberellic acid, ZR: zeatin riboside, ABA: abscisic acid

The mass fractions of endogenous hormones differed significantly among hormone types in the same bamboo organs (Table 1). Overall, ABA and IAA levels were significantly higher than those of GA and ZR. Additionally, ABA mass fractions were significantly higher than IAA mass fractions in the aboveground organs of maternal bamboo (e.g., stems, branches, and leaves), but not in belowground organs (e.g., roots). ABA and IAA mass fractions did not differ significantly in rhizomes and the sheaths of winter shoots, but ABA mass fractions were significantly higher in shoot buds, spring shoots, and winter shoots. GA and ZR mass fractions did not differ significantly in all organs sampled. Higher ABA mass fractions have been shown to enhance cold tolerance in plants [20]. Shoot temperature during the sprouting period was low, and ABA mass fractions in the aboveground organs and bamboo shoots, which were susceptible to cold damage, were correspondingly high. However, there were several winter shoots growing, and several new shoots germinating from each rhizome. GA facilitates the breaking of plant dormancy [21]. Thus, the GA mass fractions in the rhizomes were high to break rhizome dormancy and promote shoot germination.

The mass fractions of different types of endogenous hormones also varied among different organs of the same bamboo part. Among maternal bamboo organs, IAA mass fractions were highest in branches, followed by stems, roots, and leaves. GA mass fractions were highest in roots, followed by leaves, stems, and branches. ZR mass fractions were highest in roots, followed by stems, leaves, and branches. ABA mass fractions were highest in leaves, followed by branches, stems, and roots. In the rhizomes, IAA mass fractions were highest in roots, followed by latent buds, internodes, and joints. GA mass fractions were highest in roots, followed by latent buds, joints, and internodes. ZR mass fractions were highest in roots, followed by joints, internodes, and latent buds. ABA mass fractions were highest in joints, followed by internodes, latent buds, and roots. In shoots, IAA mass fractions were highest in shoot buds, followed by winter shoots, spring shoots, and the sheaths of winter shoots. GA mass fractions were highest in winter shoots, followed by sheaths, shoot buds, and spring shoots. ZR mass fractions were highest in spring shoots, followed by shoot buds, winter shoots, and sheaths. ABA mass fractions were highest in winter shoots, followed by spring shoots, sheaths, and shoot buds.

The abundances of each endogenous hormone did not differ significantly among organs of the same bamboo part, except for ZR mass fractions being higher in shoot buds and springs shoots than in other organs of the bamboo shoot. The bamboo forest community was likely dormant during the shoot-sprouting period, and bamboo metabolic and growth rates were not high. Metabolic rates do not differ substantially among organs of the same bamboo part. Thus, each endogenous hormone is distributed relatively evenly among organs. However, a greater ZR abundance in shoot buds and winter shoots can facilitate sprouting and growth, respectively.

#### 3.1.2. Shoot-growing period

The mass fractions of each endogenous hormone in the maternal bamboo organs, rhizomes, and shoots during the shoot-growing period are displayed in Table 2.

**Table 2:** Mass fractions of endogenous hormones in different bamboo parts during the shoot-growing period.

Bamboo part	Organ	IAA (ng·g^−1^)	GA (ng·g^−1^)	ZR (ng·g^−1^)	ABA (ng·g^−1^)
Maternal organs	Leaf	91.59±4.61 Bb	8.97±0.5 Ba	15.75±0.41 Ba	170.82±24.61 Bc
	Branch	79±7.64 ABb	8.65±0.13 ABa	11.48±2.4 Aa	162.28±32.24 ABc
	Stem	70.54±10.65 Ab	8.03±0.33 Aa	10.01±1.52 Aa	132.49±7.64 ABc
	Root	70.18±9.15 Ab	8.37±0.28 ABa	15.56±1.06 Ba	124.34±5.68 Ac
Rhizomes	Rhizome internode	70.40±19.42 Ab	8.73±0.98 Ba	10.02±2.16 Aa	80.25±1.45 Ab
	Rhizome joint	72.71±17.54 Ab	8.32±0.33 ABa	9.56±2.42 Aa	83.86±13.02 Ab
	Rhizome root	51.36±16.60 Ab	6.26±1.03 Aa	6.36±1.35 Aa	54.47±22.77 Ab
	Rhizome bud	55.04±10.51 Ab	6.81±1.78 ABa	8.79±2.87 Aa	70.78±39.36 Ab
Shoots	Sheath of spring shoot	89.17±30.49 Bb	7.61±2 Aa	15.56±4.74 Ba	81.51±38.49 Bb
	Shoot tip	44.64±9.23 Ac	9±2.73 Aa	17.36±5.8 Bab	29.93±8.64 Aa
	Shoot center	42.9±4.1 Ac	6.37±0.55 Aa	8.02±2.14 Aa	37.18±0.08 Ab
	Shoot base	40.02±13.42 Ab	5.69±0.79 Aa	7.34±3 Aa	32.76±8.75 Ab
	Shoot root	37.08±11.62 Ab	5.79±1.53 Aa	6.07±0.8 Aa	36.82±3.91 Ab

Note: Different uppercase letters represent significant differences (p < 0.05) among the different organs of the same bamboo part. Different lowercase letters represent significantly different levels (p < 0.05) among hormone types in the same organ. IAA: indole-3-acetic acid, GA: gibberellic acid, ZR: zeatin riboside, ABA: abscisic acid

The mass fractions of endogenous hormones were significantly different among bamboo parts (Table 2), and different from those measured during the shoot-sprouting period. In rhizomes and maternal bamboo organs, ABA was the most abundant endogenous hormone, followed by IAA, GA, and ZR. In shoots, IAA was the most abundant hormone, followed by ABA, ZR, and GA. In all organs, ABA and IAA mass fractions were significantly higher than ZR and GA mass fractions. Maternal bamboo organs had higher ABA than IAA mass fractions, and shoots and shoot centers had significantly higher IAA than ABA mass fractions. No significance differences between ABA and IAA mass fractions were found in the rhizomes. During this period, the bamboo forest community was no longer dormant, and growth and metabolic rates were high. Nutrients and rapid cell division were required to support the fast growth rates of large numbers of bamboo shoots. Therefore, bamboo shoots contained more IAA than ABA. ZR mass fractions were significantly higher in the fastest-growing shoot tips than in shoot centers and bases, whereas GA mass fractions did not differ significantly among shoot parts. Our results indicate that growth hormones were inhibited and cell division was promoted in bamboo shoots at the start of the rapid-growth period, and that growth in shoot tips was driven by cell division.

The mass fractions of each endogenous hormone also varied among organs of the same bamboo part (Table 2). Each hormone had different variation patterns depending on bamboo part, organ growth, and metabolic activity in each bamboo part. In maternal bamboo organs, IAA and ABA mass fractions were highest in leaves, followed by branches, stems, and roots. IAA mass fractions were significantly higher in leaves than in stems and roots, whereas ABA mass fractions were significantly higher in leaves than in roots. GA mass fractions were highest in leaves, followed by branches, roots, and stems, and significantly higher in leaves than stems. ZR mass fractions were highest in leaves, followed by roots, branches, and stems, and significantly higher in leaves and roots than in branches and stems. In rhizomes, IAA and ABA mass fractions were highest in rhizome joints, followed by internodes, buds, and roots, but the differences were not significant. GA and ZR mass fractions were highest in internodes, followed by joints, buds, and roots, and GA mass fractions were significantly higher in internodes than in roots. In bamboo shoots, IAA mass fractions were highest in the sheaths of spring shoots, followed by shoot tips, centers, bases, and roots, and significantly higher in the sheaths of spring shoots than in shoot tips and roots. GA mass fractions were highest in shoot tips, followed by sheaths, shoot centers, roots, and bases, but the differences were not significant. ZR mass fractions were highest in shoot tips, followed by sheaths, shoot centers, bases, and roots, and significantly higher in shoot tips and sheaths than in shoot centers, bases, and roots. ABA mass fractions were highest in the sheaths of spring shoots, followed by shoot centers, roots, bases, and tips, and significantly higher in sheaths than in other shoot parts. During this period, growth rates were higher in the aboveground part of the bamboo forest than in the belowground part. Accordingly, significantly more growth-promoting hormones were measured in the aboveground bamboo organs than in the belowground organs.

#### 3.1.3. Period of new-bamboo formation

The mass fractions of each endogenous hormone in maternal bamboo organs, rhizomes, and new bamboo plants during the period of new-bamboo formation are displayed in Table 3.

**Table 3:** Mass fractions of endogenous hormones in different bamboo parts during the period of new-bamboo formation.

Bamboo part	Organ	IAA (ng·g^−1^)	GA (ng·g^−1^)	ZR (ng·g^−1^)	ABA (ng·g^−1^)
Maternal organs	Leaf	77.19±13.43 Bb	6.86±0.76 Aa	10.53±0.9 Ba	167.13±14.08 Cc
	Branch	61.72±4.35 ABb	6.56±1.1 Aa	7.96±0.22 Aa	131.77±28.38 BCc
	Stem	64.91±8.89 ABb	5.87±0.5 Aa	7.49±1.76 Aa	119.85±23.17 ABc
	Root	52.11±3.91 Ab	6.16±0.86 Aa	8.39±1.36 ABa	91.92±8.4 Ac
Rhizomes	Rhizome internode	71.97±8.93 Ac	7.79±0.91 Aa	9.42±2.02 Aa	60.59±14.12 Ab
	Rhizome joint	67.59±7.14 Ab	7.06±1.40 Aa	9.07±2.06 Aa	91.30±20.62 Ac
	Rhizome root	79.77±8.72 Ac	7.55±0.91 Aa	10.30±1.4 Aa	66.07±15.13 Ab
	Rhizome bud	67.75±17.26 Ab	7.06±1.17 Aa	9.72±1.85 Aa	78.50±32.05 Ab
New bamboo plants	Leaf	71.90±9.42 Ab	5.86±0.42 Aa	9.48±1.10 Aa	159.05±1.75 Cc
	Branch	58.2±10.95 Ab	7.05±0.58 Aa	8.05±0.48 Aa	75.8±6.44 Bb
	Stem	72.11±10.04 Ab	7.66±0.57 Aa	7.84±0.28 Aa	53.46±7.73 ABb
	Root	70.12±11.59 Ac	5.94±0.62 Aa	8.30±1.19 Aa	37.88±1.24 Ab

Note: Different uppercase letters represent significant differences (p < 0.05) among the different organs of the same bamboo part. Different lowercase letters represent significantly different levels (p < 0.05) among hormone types in the same organ. IAA: indole-3-acetic acid, GA: gibberellic acid, ZR: zeatin riboside, ABA: abscisic acid

Across all bamboo parts, ABA was the most abundant endogenous hormone during the period of new-bamboo formation, followed by IAA, ZR, and GA (Table 3). ABA mass fractions were significantly higher than IAA mass fractions, whereas IAA mass fractions were significantly higher than ZR and GA mass fractions. Differences between ZR and GA mass fractions were not significant. In rhizome internodes and roots, IAA was most abundant, followed by ABA, ZR, and GA. IAA mass fractions were significantly higher than ABA mass fractions, whereas ABA mass fractions were significantly higher than ZR and GA mass fractions. ZR and GA mass fractions did not differ significantly. In rhizome joints and buds, ABA was most abundant, followed by IAA, ZR, and GA. In rhizome joints, ABA mass fractions were significantly higher than IAA mass fractions, whereas IAA mass fractions were significantly higher than ZR and GA mass fractions. In rhizome buds, ABA and IAA mass fractions were significantly higher than ZR and GA mass fractions. In new bamboo leaves and branches, ABA was most abundant, followed by IAA, ZR, and GA. In new bamboo stems and roots, IAA was most abundant, followed by ABA, ZR, and GA. ABA and IAA mass fractions in these four organs were significantly higher than ZR and GA mass fractions. No significant differences were found between ABA and IAA mass fractions or between ZR and GA mass fractions.

Each endogenous hormone varied in abundance among the different organs of the same bamboo part. In maternal bamboo, IAA was most abundant in leaves, followed by stems, branches, and roots. IAA mass fractions were significantly higher in branches and leaves than roots. GA was most abundant in leaves, followed by branches, roots, and stems, but the differences were not significant. ZR was most abundant in leaves, followed by roots, branches, and stems. ZR mass fractions were significantly higher in leaves than in branches and stems. ABA was most abundant in leaves, followed by branches, stems, and roots. ABA mass fractions were significantly higher in leaves than in stems and roots. In rhizomes, IAA was most abundant in roots, followed by internodes, buds, and joints. GA was most abundant in internodes, followed by roots, and then joints and buds. ZR was most abundant in roots, followed by buds, internodes, and joints. ABA was most abundant in joints, followed by buds, roots, and internodes, but the differences were not significant. In new bamboo plants, IAA was most abundant in stems, followed by leaves, roots, and branches. GA was most abundant in stems, followed by branches, roots, and leaves. ZR was most abundant in leaves, followed by roots, branches, and stems. ABA was most abundant in leaves, followed by branches, stems, and roots. Only the differences in ABA mass fractions were significant.

We speculate that, after leaf development in new bamboo plants in June, aboveground plant growth intensifies more than belowground plant growth. Rhizome roots grow quickly, and overall metabolic rates are high, and large amounts of cellulose and lignin are synthesized in stems and branches. This could explain the higher abundances of growth-promoting hormones in rhizome roots and new bamboo stems and branches at this stage. Significantly less ABA was also present in maternal bamboo roots than in aboveground organs. ABA is mainly synthesized in leaves [22]; therefore, more ABA was found in bamboo leaves than in other organs. Finally, the bamboo plants had just shed their old leaves and photosynthetic rates in the new leaves were high. Hence, IAA and ZR were more abundant in leaves than in other organs.

### 3.2. Changes in endogenous hormone levels during bamboo shooting in *P*. *edulis* ‘Pachyloen’

#### 3.2.1. Changes in endogenous hormone levels in maternal bamboo organs

**Fig 2:**
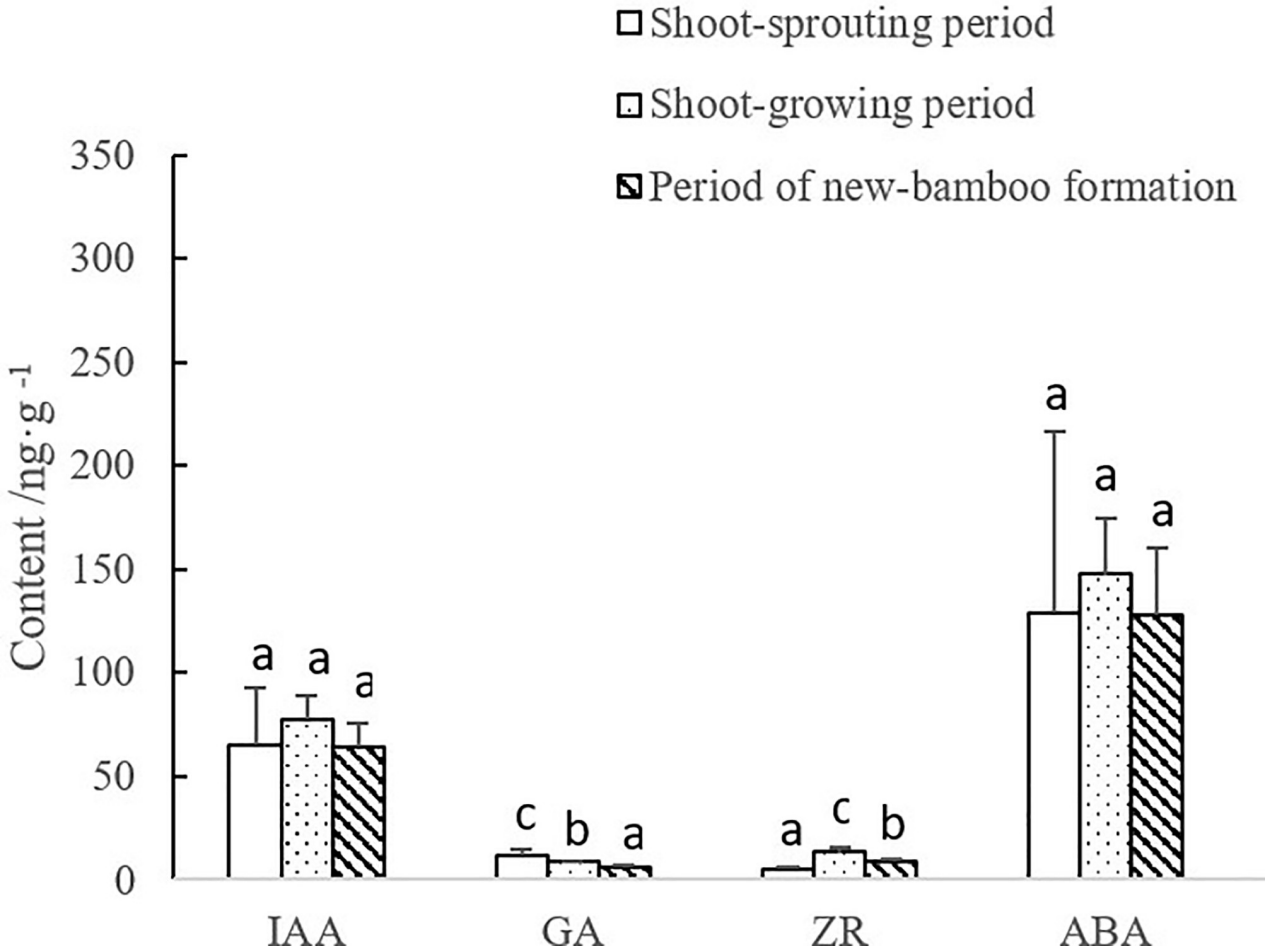
Changes in endogenous hormone levels in maternal bamboo organs over bamboo developmental periods. IAA, indole-3-acetic acid; GA, gibberellic acid; ZR, zeatin riboside; ABA, abscisic acid. Lowercase letters represent significant differences (p < 0.05) in the levels of each hormone among developmental periods.

Across the periods of shoot sprouting, shoot growth, and new-bamboo formation, IAA, ZR, and ABA mass fractions in maternal bamboo organs followed a parabolic trend, whereas GA mass fractions decreased gradually. Differences between GA and ZR abundance were significant, but differences between IAA and ABA abundance were not (Fig 2). During the shoot-sprouting period, temperatures are low and maternal bamboo plants are dormant. Therefore, GA mass fractions increased significantly in maternal bamboo organs to break plant dormancy. During the shoot-growing period, ZR mass fractions increased significantly in maternal bamboo organs to promote cell division and rapid shoot growth. After new bamboo plants emerge, growth takes place more actively in the belowground “whip” system than in the aboveground bamboo. The maternal bamboo provides organic matter to the rhizomes for growth, and largely maintain its respiratory metabolism through photosynthesis. Thus, cell division and elongation rates in maternal bamboo organs are low, with correspondingly low levels of GA and ZR.

**Fig 3:**
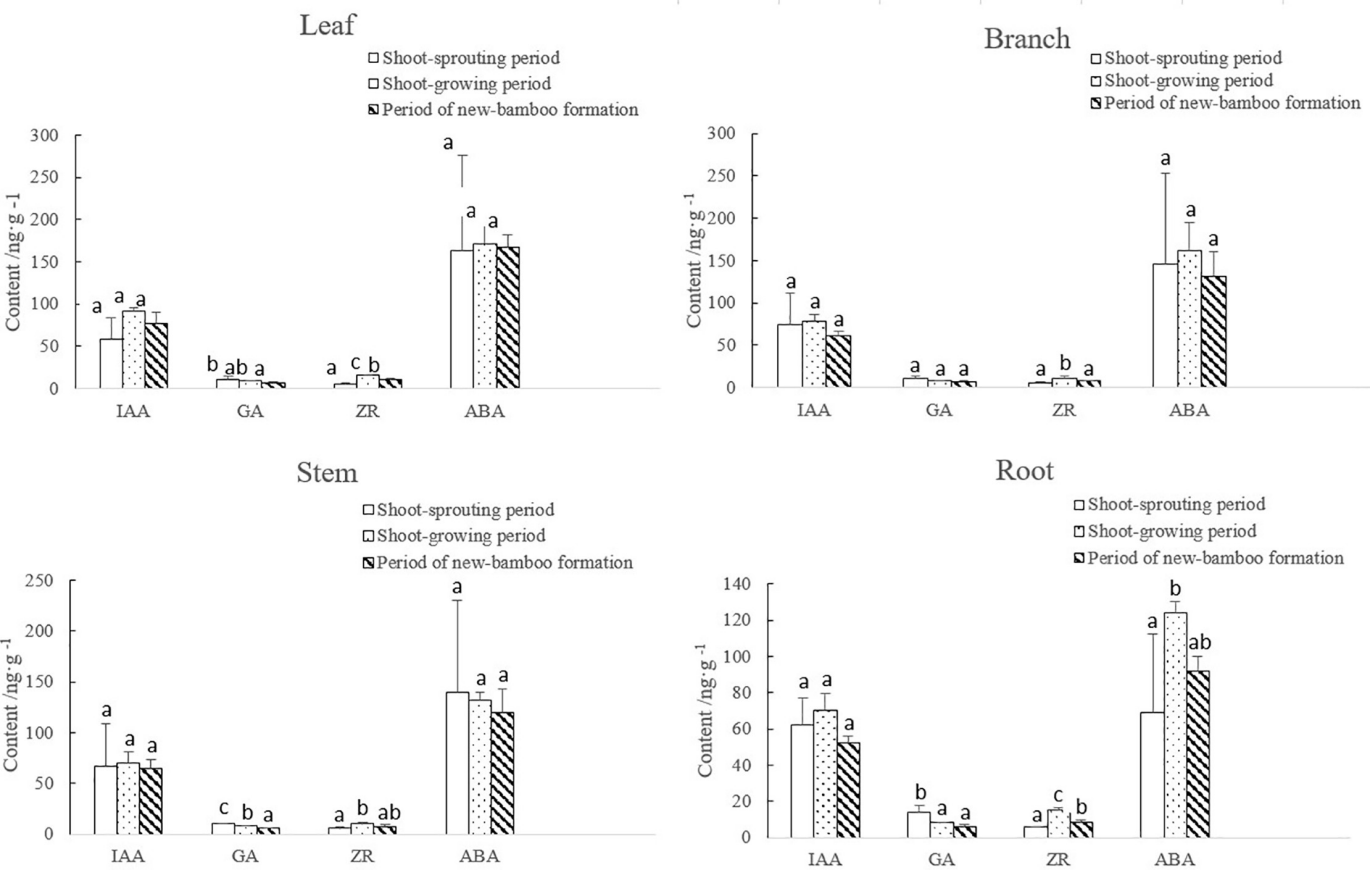
Changes in endogenous hormone levels in each maternal bamboo organ over bamboo developmental periods. IAA, indole-3-acetic acid; GA, gibberellic acid; ZR, zeatin riboside; ABA, abscisic acid. Lowercase letters represent significant differences (p < 0.05) in the levels of each hormone among developmental periods.

During the entire process of bamboo shooting, levels of growth-promoting hormones were consistent in the leaves, branches, stems, and roots of maternal bamboo plants, with IAA and ZR abundance following a parabolic trend, and GA abundance decreasing gradually in all organs (Fig 3). ZR mass fractions in all four organs were significantly higher in the shoot-growing than the shoot-sprouting period. GA mass fractions were significantly higher in the leaves, stems, and roots in the shoot-sprouting than the shoot-growing period. No significant differences in GA abundance were observed in the branches. IAA abundance in all four organs did not change significantly between periods. ABA mass fractions in leaves, branches, and roots followed a parabolic trend, whereas stem levels decreased gradually over time. Overall, levels of endogenous hormones did not differ significantly between leaves, branches, and stems, but were significantly higher during shoot growth than shoot sprouting in roots.

#### 3.2.2. Changes in endogenous hormone levels in rhizomes

**Fig 4:**
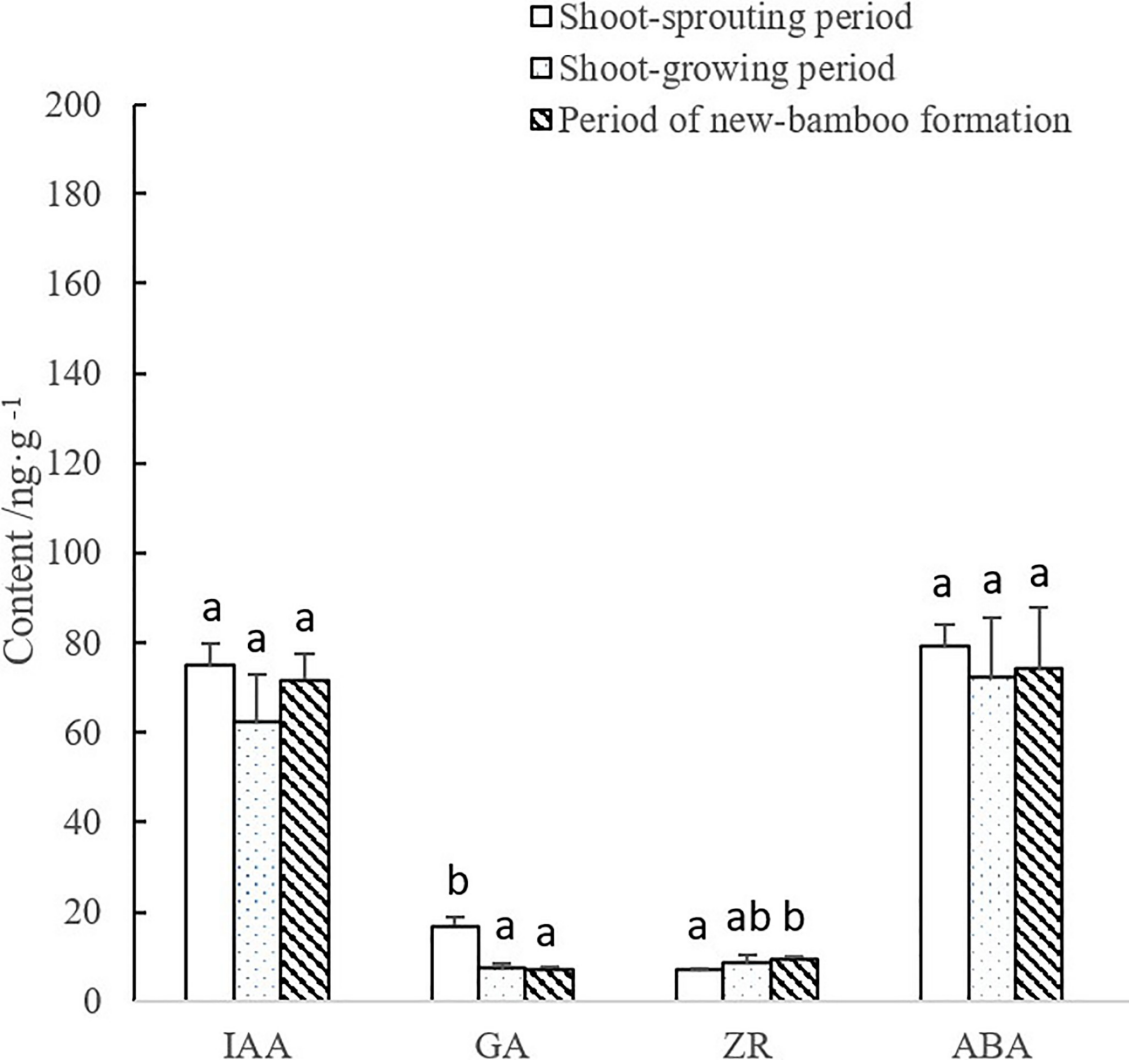
Changes in endogenous hormone levels in the rhizomes over bamboo developmental periods. IAA, indole-3-acetic acid; GA, gibberellic acid; ZR, zeatin riboside; ABA, abscisic acid. Lowercase letters represent significant differences (p < 0.05) in the levels of each hormone among developmental periods.

During shoot growth, GA mass fractions in rhizomes decreased rapidly initially, and then remained stable. GA abundance was significantly higher in the shoot-sprouting period. ZR mass fractions increased gradually over time, and levels were significantly lower during shoot sprouting than during shoot growth and new-bamboo formation. IAA and ABA mass fractions decreased at the beginning of the sampling period, then increased slightly later, but these changes were not significant (Fig 4).

**Fig 5:**
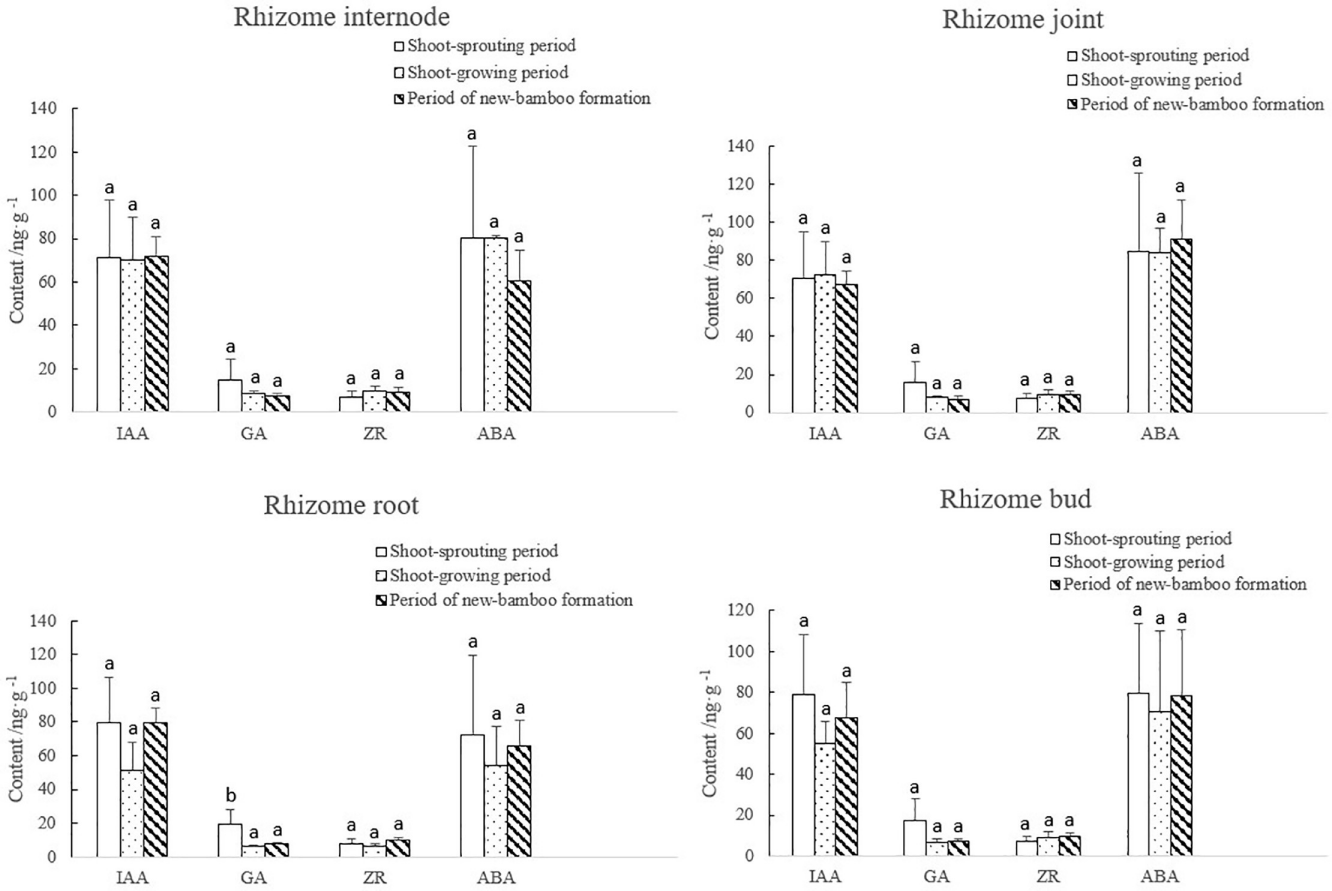
Changes in endogenous hormone levels in each rhizome organ over bamboo developmental periods. IAA, indole-3-acetic acid; GA, gibberellic acid; ZR, zeatin riboside; ABA, abscisic acid. Lowercase letters represent significant differences (p < 0.05) in the levels of each hormone among developmental periods.

The changes in hormone levels in each rhizome organ over the bamboo developmental periods are displayed in Fig 5. During the entire process of bamboo shooting, IAA mass fractions in the rhizome internodes and joints remained stable. IAA mass fractions initially decreased in the rhizome roots and buds, but these changes were not significant. GA mass fractions in all four organs decreased rapidly initially, and then remained stable. Significantly more GA was measured in the roots during shoot sprouting than during shoot growth and new-bamboo formation. ZR mass fractions increased gradually over time in all four organs, but these increases were small. ABA mass fractions did not change initially, and then decreased in the rhizome internodes. In the rhizome joints, ABA mass fractions were also stable initially, but increased later. ABA mass fractions decreased initially in the rhizome roots and buds, and then increased, but these differences were not significant. The levels of each endogenous hormone exhibited different temporal trends in rhizomes, indicating that hormone levels corresponded with growth activities within the bamboo plant to effectively regulate the growth and development of different organs. During the shoot-sprouting period, GA levels in the rhizomes were significantly higher to break plant dormancy and to promote latent bud sprouting and winter shoot growth from the rhizomes. After plant dormancy was broken, GA mass fractions decreased significantly. During shoot growth, bamboo shoots exhibit the highest growth and metabolic rates, and maternal bamboo plants actively photosynthesize and have high metabolic rates. In contrast, growth and metabolic rates in rhizome roots and buds are low. Thus, we expect that auxin levels decrease in rhizome roots and buds, and remain low in rhizome internodes and joints, which are involved in transport and storage. After new bamboo leaves have unfolded, growth rates would be higher in the rhizomes. At this time, rhizome buds sprout into new rhizomes that grow rapidly, and existing rhizome roots grow quickly. Thus, levels of endogenous hormones would increase in the rhizome roots and buds.

#### 3.2.3. Changes in endogenous hormone levels during new-bamboo formation

**Fig 6:**
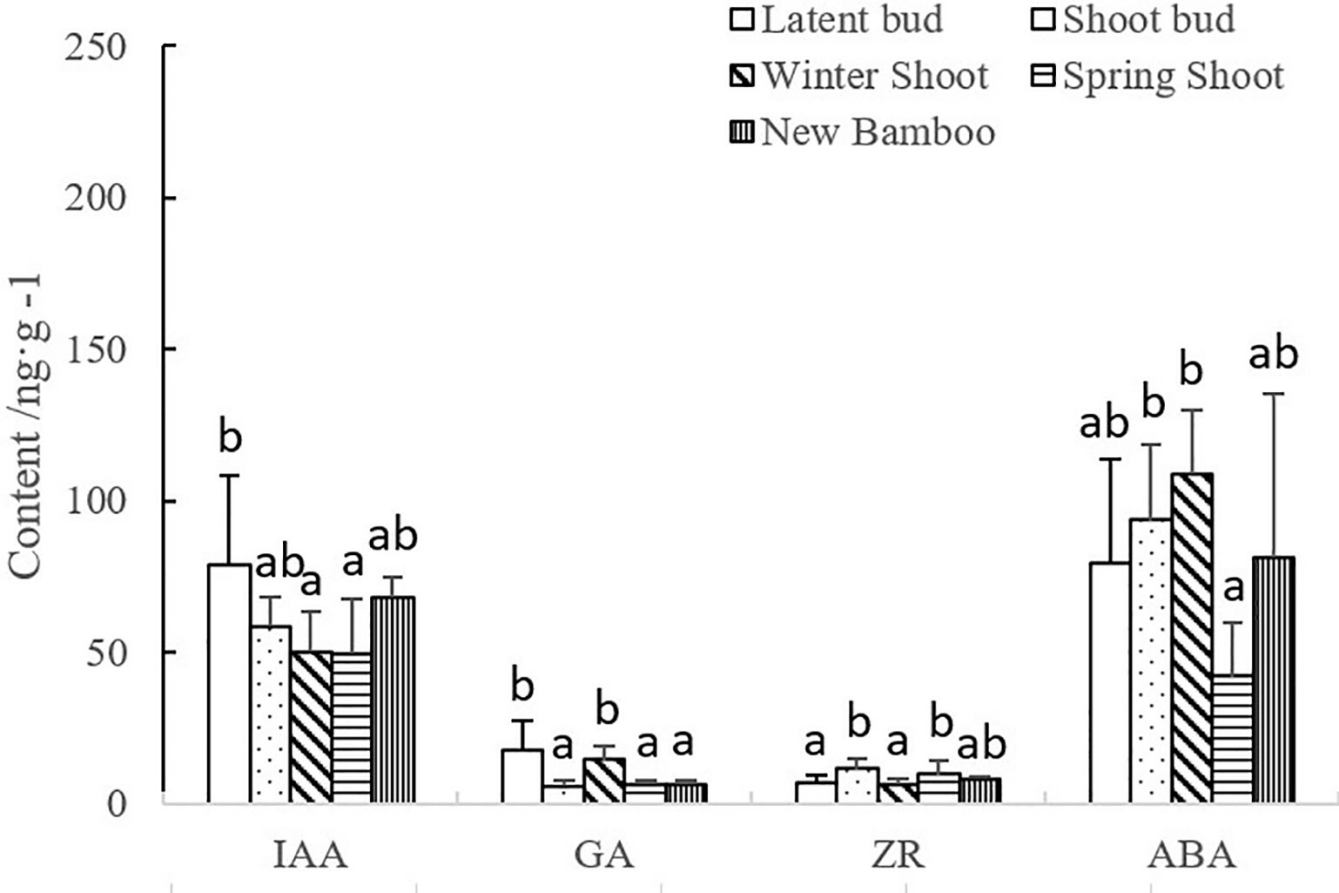
Changes in endogenous hormone levels in different bamboo parts during the period of new-bamboo formation. IAA, indole-3-acetic acid; GA, gibberellic acid; ZR, zeatin riboside; ABA, abscisic acid. Different lowercase letters represent significant differences (p < 0.05) in the levels of each hormone among bamboo parts.

The levels of endogenous hormones in latent buds, shoot buds, winter shoots, spring shoots, and new bamboo plants are displayed in Fig 6.

The variations in each endogenous hormone among bamboo parts differed for each hormone type (Fig 6). IAA abundance was significantly lower in winter shoots than in latent buds, and lowest during the formation of winter and spring shoots. IAA abundance increased again, significantly, during new-bamboo formation. GA mass fractions decreased significantly during shoot differentiation, increased significantly during shoot sprouting, and decreased significantly again when spring shoots emerged through the soil. ZR levels followed a trend opposite to that of GA levels. ABA mass fractions increased significantly during gestation, peaked in winter shoots, and then decreased rapidly. The lowest ABA mass fractions were measured when spring shoots were growing, and levels started increasing after shoots emerged. Our results indicate that the latent buds on the rhizomes contributed to the breaking of dormancy and promoted the differentiation of shoot buds. Hence, GA and IAA levels were higher in latent buds compared to the other bamboo parts. As shoot buds differentiated, GA mass fractions decreased significantly. Cell division is the dominant process during shoot-bud differentiation; thus, ZR levels increased significantly. As temperatures decrease, shoot buds develop into winter shoots, which then remain dormant. Cell division rates in the winter shoots would decrease, and the ZR levels in our sample also decreased significantly. To tolerate the low temperatures and break dormancy, ABA and GA levels increased significantly in winter shoots. In late March, temperatures increase, and winter shoots are no longer dormant. Winter shoots then develop into spring shoots and start growing rapidly. In tandem with the fast cell division in spring shoots, ZR mass fractions also increased significantly, and GA and ABA mass fractions decreased significantly.

### 3.3. Ratios of endogenous hormones during bamboo shooting

Hormones can interact synergistically or antagonistically to affect bamboo plants [13]. Thus, changes in the ratios of endogenous hormones during bamboo shooting in *P*. *edulis* ‘Pachyloen’ may reflect the synergistic or antagonistic effects of the hormones on the regulation of bamboo shooting.

#### 3.3.1. Ratios of endogenous hormones at different developmental periods

The ratios of the endogenous hormones measured in this study were compared between the rhizomes, shoots, and maternal bamboo organs at different developmental periods, and among developmental periods in the same bamboo part. The results are summarized in Table 4. During the shoot-sprouting period, the bamboo forest is dormant, and growth and metabolic rates are low. Therefore, the ratios of endogenous hormones were mostly similar in the rhizomes, winter shoots, and maternal bamboo organs. However, ZR: IAA and GA: IAA ratios were significantly higher in winter shoots than in rhizomes and maternal bamboo organs, indicating that higher proportions of ZR and GA, which are growth-promoting hormones, contributed to the development of winter shoots and breaking of dormancy. During the shoot-growing period, the ratios of growth-promoting hormones (i.e., IAA, ZR, and GA) to ABA, a growth-inhibiting hormone, were significantly higher in spring shoots than in rhizomes and maternal bamboo organs. ZR: GA and ZR: IAA ratios were significantly higher in spring shoots than in rhizomes, and the GA: IAA ratio was significantly higher in spring shoots than in maternal bamboo organs. As temperatures rise, spring shoots are expected to have the highest growth rates within the bamboo forest. At this time, the ZR: GA ratio was significantly higher in maternal bamboo organs than in rhizomes, whereas the IAA: ABA, GA: ABA, and (IAA+ZR+GA): ABA ratios were significantly lower in maternal bamboo organs than in rhizomes. After new bamboo leaves develop, plant growth becomes more intensive in the belowground component of the bamboo forest. Most of the energy for growth goes towards the differentiation of rhizome buds and rhizome growth. Accordingly, we found that there was a significantly higher proportion of growth-promoting hormones (IAA, ZR, GA) in new bamboo plants than in the rhizomes and maternal bamboo organs.

**Table 4:** Ratios of endogenous hormones at different developmental periods.

Developmental period	Bamboo part	IAA: ABA	GA: ABA	ZR: ABA	(IAA+ZR+GA): ABA	ZR: GA	ZR: IAA	GA: IAA
Shoot-sprouting period	Rhizome	1.30±0.94A	0.29±0.28 A	0.12±0.10 A	1.71±1.24 A	0.49±0.18A	0.10±0.03A	0.23±0.10A
	Winter shoot	0.91±1.00A	0.24±0.30 A	0.11±0.14 A	1.26±1.40 A	0.46±0.77A	0.14±0.49B	0.31±0.11B
	Maternal bamboo	0.78±0.64A	0.15±0.13 A	0.07±0.05 A	1.00±0.80 A	0.51±0.16A	0.09±0.34A	0.20±0.08A
Shoot-growing period	Rhizome	0.93±0.37B	0.11±0.03B	0.13±0.03 A	1.17±0.42 B	1.18±0.21A	0.14±0.02A	0.13±0.04AB
	Spring shoot	1.22±0.31C	0.18±0.08 C	0.28±0.20 B	1.69±0.55 C	1.53±0.55B	0.22±0.09B	0.15±0.05B
	Maternal bamboo	0.53±0.07A	0.06±0.00 A	0.09±0.02 A	0.68±0.09 A	1.55±0.32B	0.17±0.04AB	0.11±0.01A
Period of new-bamboo formation	Rhizome	1.09±0.43B	0.11±0.04B	0.15±0.06 B	1.34±0.53 B	1.32±0.22A	0.14±0.02A	0.10±0.02A
	New bamboo	1.13±0.67B	0.11±0.06B	0.13±0.07 B	1.37±0.78 B	1.29±0.26A	0.13±0.03A	0.10±0.02A
	Maternal bamboo	0.52±0.12A	0.05±0.02 A	0.07±0.02 A	0.65±0.15 A	1.36±0.25A	0.14±0.02A	0.10±0.02A

Note: IAA, indole-3-acetic acid; GA, gibberellic acid; ZR, zeatin riboside; ABA, abscisic acid. Different uppercase letters represent significant differences (p < 0.05) in hormone ratios among bamboo parts within the same developmental period.

#### 3.3.2. Changes endogenous hormone ratios in maternal bamboo organs

**Fig 7:**
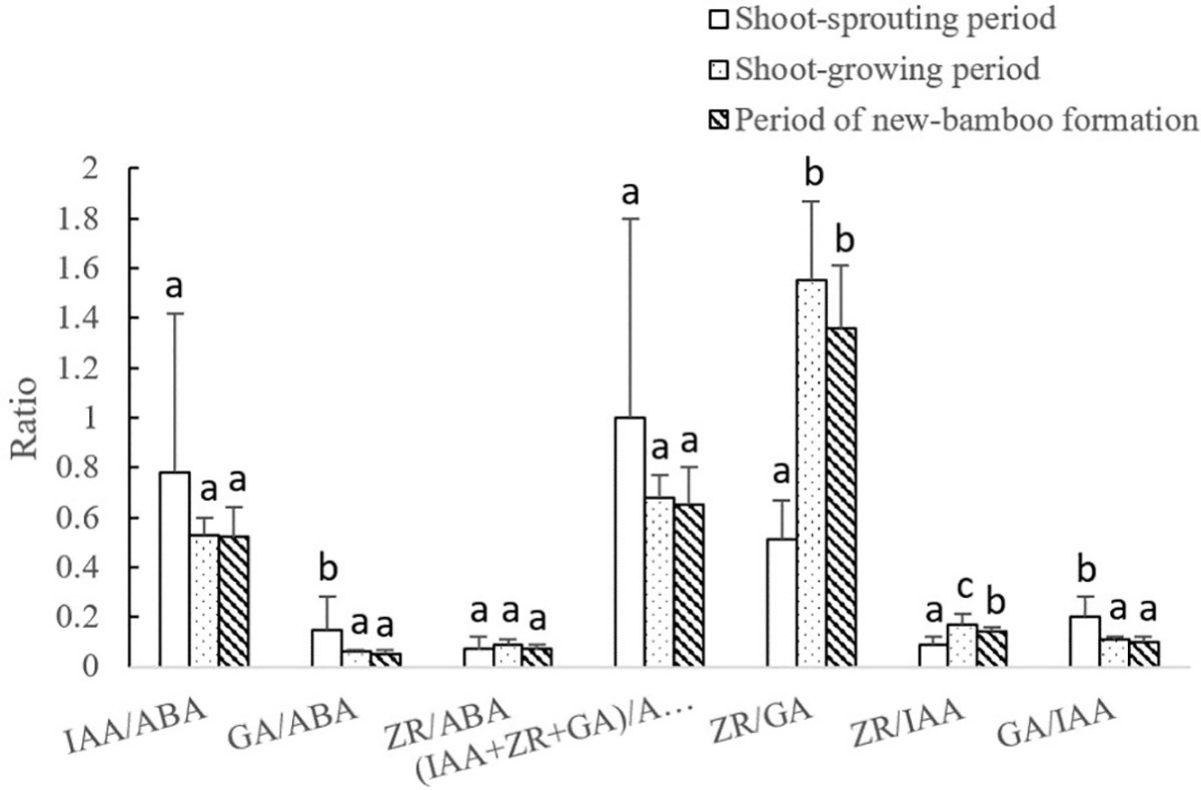
Changes in endogenous hormone ratios in maternal bamboo organs across developmental periods. IAA, indole-3-acetic acid; GA, gibberellic acid; ZR, zeatin riboside; ABA, abscisic acid. Different lowercase letters represent significant differences (p < 0.05) in each ratio among developmental periods.

The ratios of endogenous hormones varied across the entire process of bamboo shooting, but variation patterns and ranges differed among hormone ratios (Fig 7). The ratios of growth-promoting to growth-inhibiting hormones were generally small, and a parabolic trend was only observed for the ZR: ABA ratio. The other ratios decreased more sharply in the early developmental periods, and remained relatively stable thereafter. The initial decreases were small, and only the decrease in the GA: ABA ratio was significant. For the growth-promoting hormones, the ZR: GA and ZR: IAA ratios followed a parabolic trend, with significant increases initially. Thereafter, the ZR: IAA ratio decreased significantly, but not the ZR: GA ratio. Significant decreases were observed in the GA: IAA ratio initially, and values remained stable thereafter.

**Fig 8:**
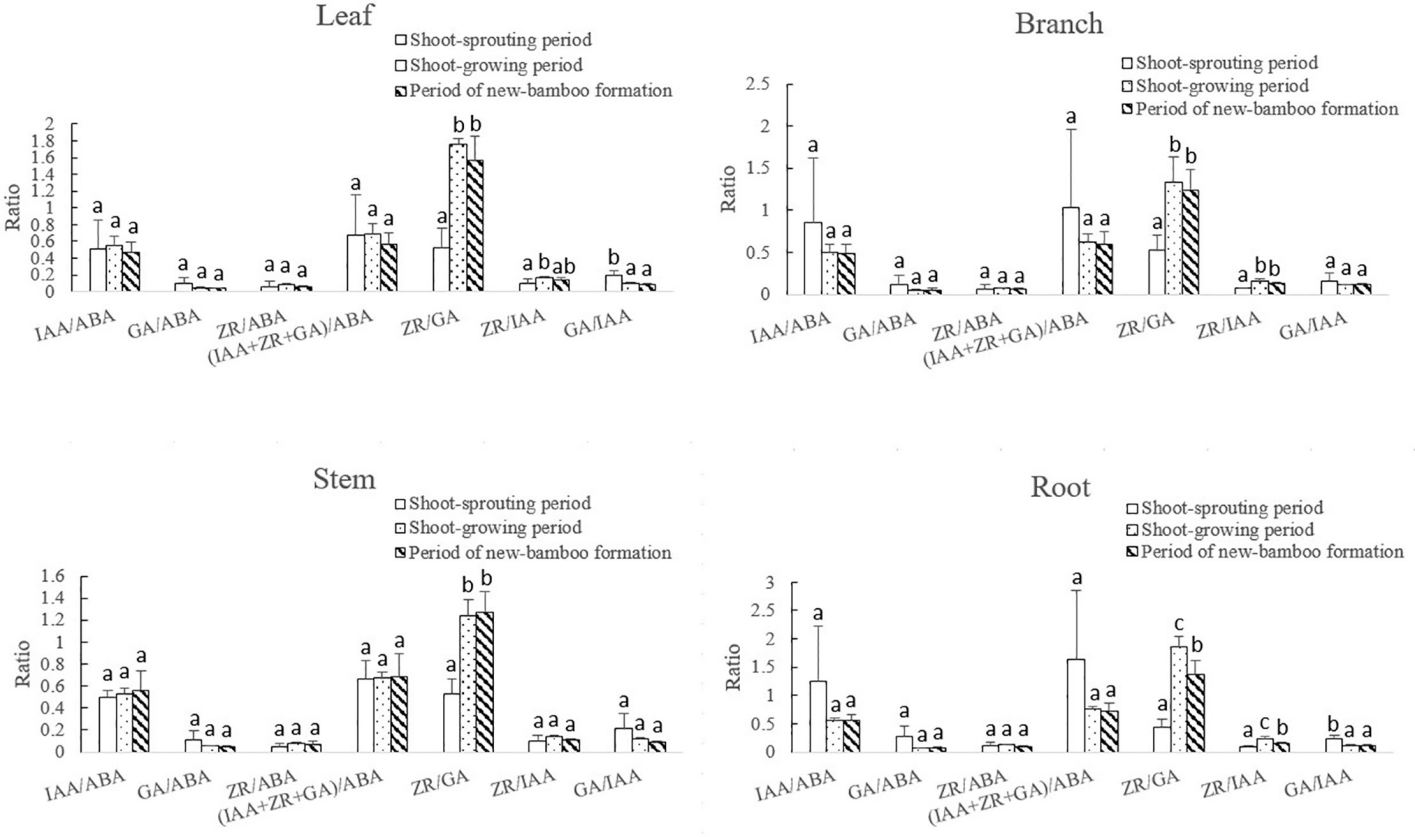
Changes in endogenous hormone ratios in each maternal bamboo organ across developmental periods. IAA, indole-3-acetic acid; GA, gibberellic acid; ZR, zeatin riboside; ABA, abscisic acid. Different lowercase letters represent significant differences (p < 0.05) in each ratio among developmental periods.

The ratios of endogenous hormone varied in a similar manner among the four maternal bamboo organs—leaves, branches, stems, and roots—across all developmental periods (Fig 8). The ratios of growth-promoting to growth-inhibiting hormones did not change significantly, though the overall difference in the ratios was large. The ZR: GA ratio increased significantly during the shoot-sprouting period. However, during shoot growth and new-bamboo formation, the ZR: GA ratio decreased significantly in roots, decreased slightly in leaves and branches, and increased slightly in stems. The ZR: IAA ratio followed a parabolic trend, and the GA: IAA ratio generally decreased over time. The largest variation in the GA: IAA ratio was found in roots, followed by leaves, branches, and stems. During the shoot-sprouting period, the ZR: GA ratio increased significantly in stems, but did not change significantly in the other organs. The results indicate that synergistic and antagonistic interactions among growth-promoting hormones significantly affect the maternal bamboo organs in *P*. *edulis* ‘Pachyloen.’ Specifically, these hormones were mainly active in roots and leaves, which have relatively high rates of growth and metabolism.

#### 3.3.3. Changes in endogenous hormone ratios in rhizomes

**Fig 9:**
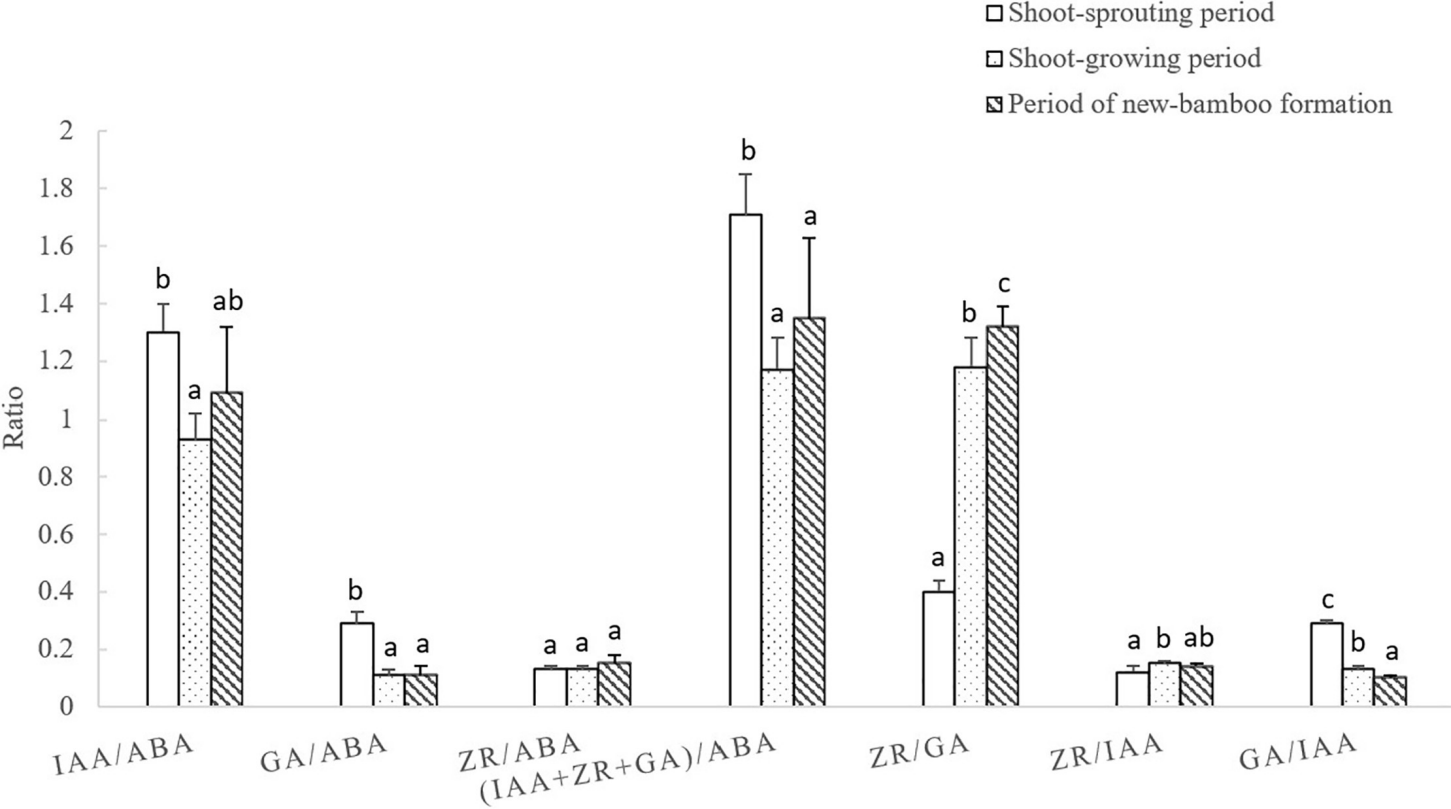
Changes in endogenous hormone ratios in bamboo rhizomes across developmental periods. IAA, indole-3-acetic acid; GA, gibberellic acid; ZR, zeatin riboside; ABA, abscisic acid. Different lowercase letters represent significant differences (p < 0.05) in each ratio among developmental periods.

The variation pattern and range of each endogenous hormone ratio differed among developmental periods (Fig 9). The ratios of growth-promoting to growth-inhibiting hormones and IAA: ABA decreased significantly during the shoot-sprouting period, and then increased slightly during shoot growth and new-bamboo formation. The GA: ABA and GA: IAA ratios decreased significantly during the shoot-sprouting period. The GA: IAA ratio decreased significantly again during shoot growth and new-bamboo formation, but the GA: ABA ratio only decreased slightly. The ZR: GA ratio increased significantly over time, whereas the ZR: IAA ratio exhibited a parabolic trend, with significantly higher values during shoot growth. The ZR: ABA ratio remained stable throughout the bamboo-shooting process.

**Fig 10:**
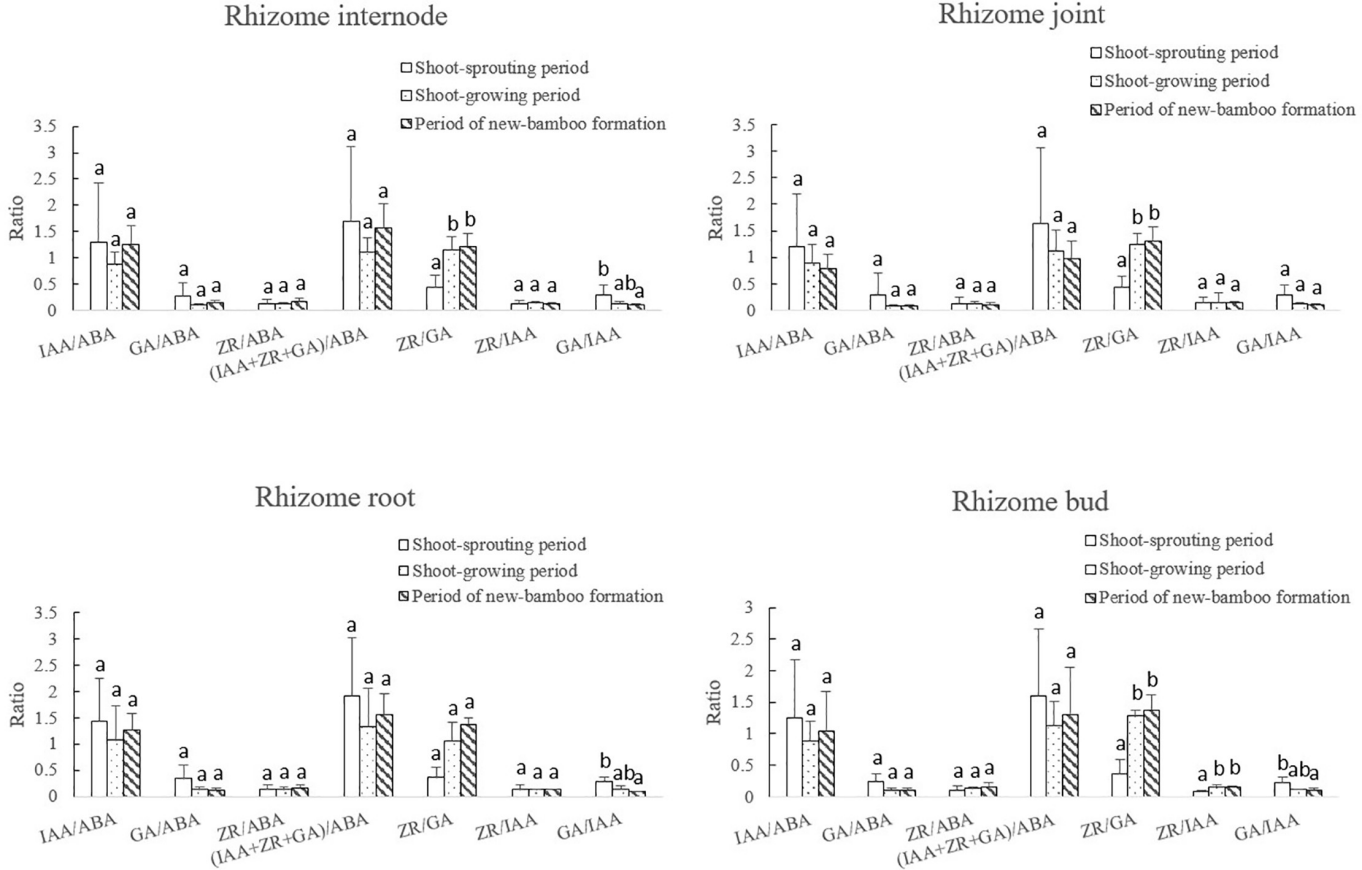
Changes in endogenous hormone ratios in each rhizome organ across developmental periods. IAA, indole-3-acetic acid; GA, gibberellic acid; ZR, zeatin riboside; ABA, abscisic acid. Different lowercase letters represent significant differences (p < 0.05) in each ratio among developmental periods.

The ZR: GA ratio in the rhizome internodes, joints, roots, and buds increased gradually over the three developmental periods (Fig 10); although it did not differ significantly among the developmental periods in roots, the ZR: GA ratio was significantly lower during shoot sprouting than during shoot growth and new-bamboo formation in internodes, joints, and buds (Fig 10). The ZR: IAA ratio increased significantly from shoot sprouting to shoot growth, followed by a slight decrease during new-bamboo formation in rhizome buds; however, it did not change significantly in internodes, joints, and roots. The GA: IAA ratio decreased gradually over time. In internodes, roots, and buds, the GA: IAA ratio was significantly higher during shoot sprouting than during new-bamboo formation; however, no significant changes were observed in joints. Significant changes were also not observed for the IAA: ABA, ZR: ABA, GA: ABA, and (IAA+ZR+GA): ABA ratios.

#### 3.3.4. Changes in endogenous hormone ratios during new-bamboo formation

**Fig 11:**
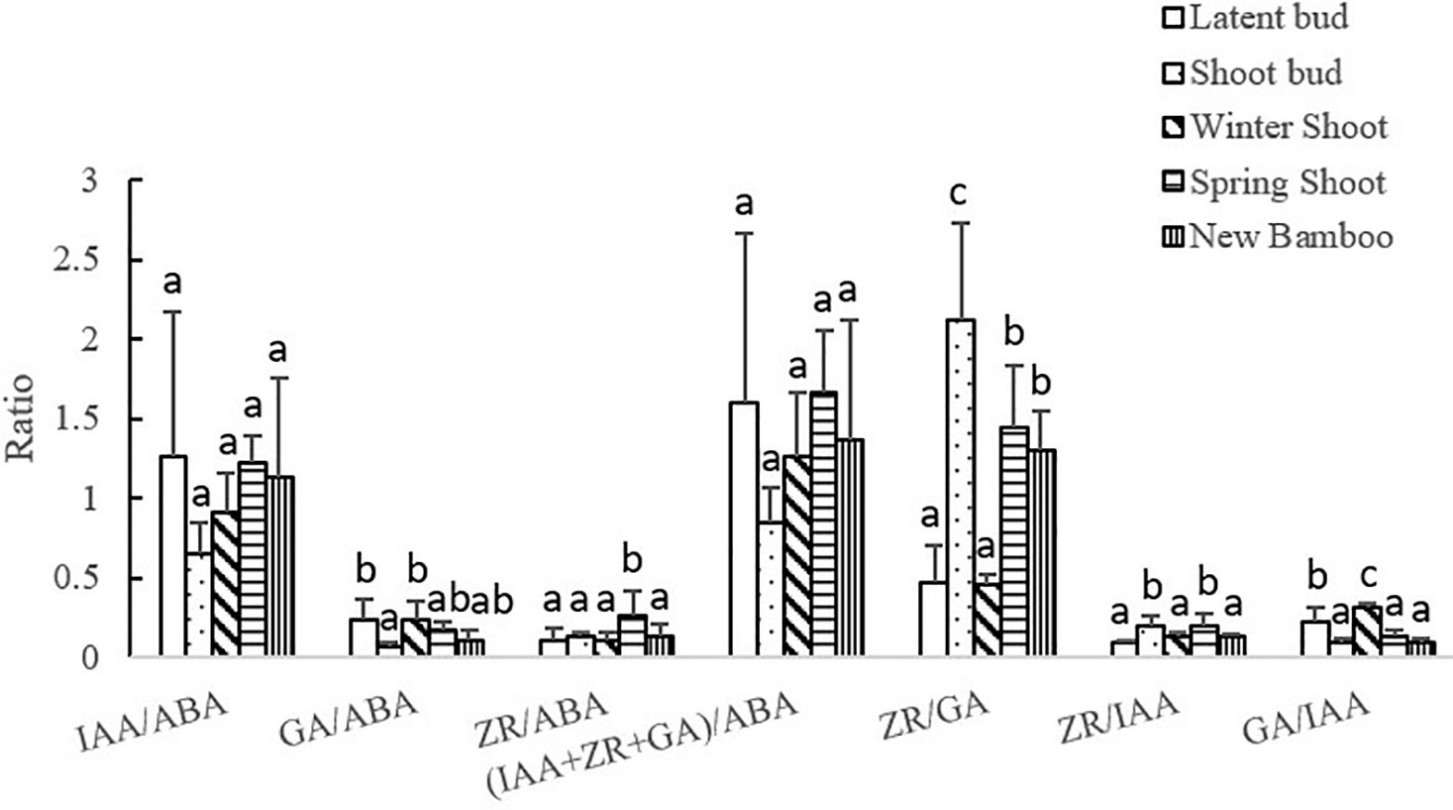
Changes in endogenous hormone ratios in bamboo parts during the period of new-bamboo formation. IAA, indole-3-acetic acid; GA, gibberellic acid; ZR, zeatin riboside; ABA, abscisic acid. Different lowercase letters represent significant differences (p < 0.05) in each ratio among bamboo parts.

Changes in endogenous hormone ratios were observed among different bamboo organs during the period of new-bamboo formation (Fig 11). GA: ABA and GA: IAA ratios were high in latent buds, significantly lower in shoot buds (bud differentiation), significantly higher again in winter shoots (shoot sprouting), significantly lower in spring shoots (shoot growth), and remained similar in new bamboo plants (leaf expansion). The lowest ZA: IAA and ZR: GA ratios were recorded in latent buds. The ratios increased significantly during bud differentiation, decreased significantly during shoot sprouting, and increased significantly during shoot growth. The ZR: IAA ratio was significantly lower during leaf expansion, but changes in the ZR: GA ratio were not significant. The ZR: ABA ratio was similar across all bamboo parts, except for spring shoots, which had a significantly higher ratio than other parts. The IAA: ABA and (IAA+ZR+GA): ABA ratios did not change significantly during the period of new-bamboo formation.

## 4. Discussion

### 4.1. Endogenous hormone levels in *P*. *edulis* ‘Pachyloen’ depend on plant growth activity

During the period of bamboo shooting, growth and metabolic activities varied among the bamboo parts [23]. In addition, growth rates peaked at different times among different bamboo parts [24]. Thus, levels of endogenous hormones had to adjust to promote the normal growth and development of the bamboo forest.

The bamboo forest is dormant during the shoot-sprouting period. At this time, significantly higher levels of growth-inhibiting hormones such as ABA were found in the winter shoots and rhizome buds instead of growth-promoting hormones [25]. This likely inhibited rapid shoot growth in low temperatures. The main functions of GA are the promotion of cell elongation and breaking of plant dormancy [21]. Accordingly, GA levels in rhizomes were approximately three times the levels during the periods of shoot growth and new-bamboo formation. ZR levels were low and GA levels were high in winter shoots. Therefore, winter shoot growth during the shoot-sprouting period may be dominated by cell elongation. IAA and GA levels increased significantly in the belowground organs of maternal bamboo. ABA levels increased, but not significantly, whereas no changes in ZR levels were observed. Maternal bamboo is dormant during this period, and may synthesize IAA and GA at low rates, but the belowground organs might still undergo active growth and cell differentiation, and thus produce large amounts of IAA and GA [26]. Some hormones can be transported in water to the aboveground organs. Hence, the mass fractions of these hormones decreased with increasing distance from the rhizomes.

During the shoot-growing period, growth rates were higher in the aboveground than belowground bamboo parts. Cell division and elongation rates are high, which corresponds to high levels of growth-promoting hormones such as ZR and GA [27]. In bamboo shoots, the ZR mass fraction increased significantly, whereas the ABA mass fraction decreased significantly. The ZR mass fraction was significantly higher in shoot tips than in shoot centers and bases, indicating that cell division is the primary growth process in the shoot tip. During this period, metabolic and synthetic rates in leaves increases to synthesize more organic matter and endogenous hormones for the growth of bamboo shoots and new bamboo plants. Thus, GA, ZR, IAA, and ABA levels decreased significantly in the belowground organs of the maternal bamboo, consistent with previous reports [12]. After the growth of new bamboo, the ZR and GA mass fractions in bamboo leaves were significantly lower than those in rhizomes, indicating that cell division and growth in aboveground parts ceased after new bamboo plants developed. The center of growth activity then moved to the belowground rhizome system of the bamboo forest.

During bamboo shooting, levels of different endogenous hormones varied within the same organ, and levels of the same hormone varied among different organs. This indicates that different endogenous hormones have different effects on growth and development in *P*. *edulis* ‘Pachyloen.’ Levels of endogenous hormones changed across the bamboo forest in accordance with the growth and developmental activities in different bamboo parts at various developmental periods. The variations in IAA, GA, and ZR in rhizomes were opposite to those in maternal bamboo leaves and branches. This is likely due to the alternation of high growth rates between belowground (rhizomes) and aboveground (maternal bamboo leaves and branches) bamboo parts during bamboo shooting.

### 4.2. Effects of synergistic and antagonistic interactions among endogenous hormones on the regulation of bamboo shooting in *P*. *edulis* ‘Pachyloen’

More than 300 types of endogenous hormones with varying physiological functions have been discovered to date [21]. However, only a few types of endogenous hormones have been studied in detail [28–30]. Synergistic and antagonistic interactions among endogenous hormones have been shown to be important in regulating the growth and development of bamboo plants. For example, the IAA: ABA ratio influences bud germination in *Phyllostachys praecox* C. D. Chu et C. S. Chao ‘Prevernalis,’ and the GA: ABA ratio is associated with the development of bamboo rhizomes and shoots [13]. In this study, the ratios of endogenous hormones varied among bamboo parts and developmental periods in *P*. *edulis* ‘Pachyloen.’ GA mass fractions relative to those of other hormones were higher in latent buds and winter shoots, whereas proportional ZR levels were higher in spring shoots. ZR: IAA, ZR: ABA, GA: IAA, and GA: ABA ratios were higher in shoot tips than in other bamboo parts. After the development of new bamboo plants, the highest growth rates are found in the belowground bamboo parts. Accordingly, the ratio of growth-promoting to growth-inhibiting hormones increased significantly in the new bamboo plants. Additionally, GA: IAA ratios were significantly lower in leaves and roots than in and branches, but ZR: GA ratios exhibited the opposite trend. These results indicate that the interactions among the endogenous hormones affect the growth and development of *P*. *edulis* ‘Pachyloen.’ Our analysis of the changes in ratios of endogenous hormones in the rhizomes and maternal bamboo organs revealed that synergistic and antagonistic interactions among hormones greatly influenced the growth, development, and formation of bamboo shoots in *P*. *edulis* ‘Pachyloen.’ Specifically, changes in ZR and GA ratios help to regulate bamboo shooting. ZR: GA, ZR: IAA, and GA: IAA ratios changed significantly during bamboo shooting, growth, and development. Higher ZR proportions could have promoted cell division to facilitate carbohydrate synthesis and organ growth [31]. Higher GA proportions are conducive to shoot-bud differentiation, winter-shoot germination, breaking the dormancy of latent buds, matter transformation, and the formation of cellulose and lignin. Changes in IAA: GA ratios also regulate bamboo shooting by regulating sprouting in rhizome buds and the development of shoot buds. Overall, the synergistic and antagonistic interactions among different endogenous hormones are important for the regulation of bamboo shooting.

### 4.3. Association between endogenous hormones and thick wall development in *P*. *edulis* ‘Pachyloen’

In the spring shoots of *Phyllostachys pubescens*, kinetin (KT) is the most abundant endogenous hormone, followed by gibberellin (GA3) and IAA [15]. In contrast, GA3 is the most abundant endogenous hormone in new leaves, followed by IAA and KT [15]. Similarly, Wang et al. [16] found that GA3 mass fractions in bamboo shoots and new leaves were significantly higher than IAA mass fractions. GA3 mass fractions were also significantly higher than zeatin mass fractions in bamboo shoots and new bamboo tissues [32]. In this study, IAA was the most abundant hormone overall, followed by ZR and GA. IAA mass fractions were also significantly higher than ZR and GA mass fractions. During the shoot-sprouting period, GA levels were higher than ZR levels, whereas ZR levels were higher than GA levels in the periods of shoot growth and new-bamboo formation. However, these differences were not significant. IAA promotes cell-wall formation and protein synthesis, ZR promotes cell division, and GA promotes cell elongation. When IAA and ZR levels are high and GA levels are low, the lateral growth of the bamboo walls may be enhanced, which leads to wall-thickening in *P*. *edulis* ‘Pachyloen.’

ZR transport in plants is regulated by plant nitrogen metabolism [33]. Nitrogen metabolic rates in *P*. *edulis* ‘Pachyloen’ are higher than in *Phyllostachys heterocycla* [5], which may explain the relatively high ZR abundance in *P*. *edulis* ‘Pachyloen.’ High ABA levels and ABA: GA ratios can help plants tolerate cold stress [17, 34]. In this study, ABA was more abundant than IAA in rhizomes and winter shoots during the dormant period. In contrast, ABA mass fractions in *P*. *heterocycla* are significantly lower than those of IAA [16]. This could partially explain why *P*. *edulis* ‘Pachyloen’ is colder tolerant than *P*. *heterocycla* [4].

## 5. Conclusion

The mass fractions of endogenous hormones in *P*. *edulis* ‘Pachyloen’ varied significantly among hormone types. Overall, levels of IAA and ABA were significantly higher than those of ZR and GA. Differences in hormone levels were driven by bamboo developmental period. During bamboo shooting, the endogenous hormones were variably distributed among maternal bamboo, rhizomes, and shoots. The endogenous hormones, and their synergistic and antagonistic interactions, have different regulatory effects on the growth and development of *P*. *edulis* ‘Pachyloen.’ In particular, the synergistic and antagonistic interactions among growth-promoting hormones strongly influenced the bamboo-shooting process. Relatively high ZR and IAA levels and relatively low GA levels might be responsible for the formation of the thick walls characteristic of *P*. *edulis* ‘Pachyloen.’

## Supporting information

S1 Data (XLSX)
